# Fenofibrate alleviates NAFLD by enhancing the PPARα/PGC-1α signaling pathway coupling mitochondrial function

**DOI:** 10.1186/s40360-023-00730-6

**Published:** 2024-01-03

**Authors:** Xuemei Wang, Jieying Wang, Cao Ying, Yuan Xing, Xuan Su, Ke Men

**Affiliations:** Department of public health, Xi’an Medical College, No. 1 Xinwang Road, Weiyang District, Xi’an, Shaanxi 710000 China

**Keywords:** Nonalcoholic fatty liver disease, Fenofibrate, Lipid accumulation, Mitochondria function, Peroxisome proliferators activated receptors gamma co-activator 1α

## Abstract

**Background:**

To comprehend the influences of fenofibrate on hepatic lipid accumulation and mitochondrial function-related signaling pathways in mice with non-alcoholic fatty liver disease (NAFLD) secondary to high-fat diets together with free fatty acids-influenced HepG2 cells model.

**Materials and methods:**

A random allocation of male 6-week C57BL/6J mice into three groups was done, including controls, model (14 weeks of a high-fat diet), and fenofibrate [similar to the model one with administered 0.04 g/(kg.d) fenofibrate by gavage at 11 weeks for 4 weeks] groups, which contained 10 mice each. This study verified NAFLD pathogenesis via mitochondrial functions in hepatic pathological abnormalities, liver index and weight, body weight, serum biochemical indexes, oxidative stress indicators, mitochondrial function indexes, and related signaling pathways. The effect of fenofibrate intervention was investigated in NAFLD model mice. In vitro, four groups based on HepG2 cells were generated, including controls, the FFA model (1.5 mmol/L FFA incubation for 24 h), LV-PGC-1α intervention (similar to the FFA model one after PPARGC1A lentivirus transfection), and LV control intervention (similar to the FFA model one after negative control lentivirus transfection) groups. The study investigated the mechanism of PGC-1α related to lipid decomposition and mitochondrial biosynthesis by Oil red O staining, colorimetry and western blot.

**Results:**

In vivo experiments, a high-fat diet achieved remarkable changes regarding liver weight, liver index, serum biochemical indicators, oxidative stress indicators, liver pathological changes, mitochondrial function indicators, and body weight of the NAFLD model mice while fenofibrate improved the objective indicators. In the HepG2 cells model, the lipid accumulation increased significantly within the FFA model group, together with aggravated hepatocytic damage and boosted oxidative stress levels. Moreover, FFA induced excessive mitosis into fragmented in mitochondrial morphology, ATP content in cells decreased, mtDNA replication fold decreased, the expression of lipid decomposition protein PPARα reduced, mitochondrial biosynthesis related protein PGC-1α, NRF-1 and TFAM decreased. PGC-1α overexpression inhibited lipid deposition by improving mitochondrial biosynthesis and lipid decomposition.

**Conclusion:**

Fenofibrate up-regulated PPARα/PGC-1α signaling pathway, promoted mitochondrial β-oxidation, reduced oxidative stress damage and lipid accumulation of liver. PGC-1α overexpression enhanced mitochondrial biosynthesis and ATP production, and reduced HepG2 intracellular accumulation of lipids and oxidative stress.

## Introduction

Without a history of heavy alcohol intake or other etiologies of liver disease, nonalcoholic fatty liver disease (NAFLD) may develop when fat accumulates in the hepatocytes to a significant degree (> 5%). Simple steatosis is a hallmark of a wide variety of liver disorders, and NAFLD comprises all of them. There may be a variety of steps in the disorder’s possible progression. For instance, approximately one-fifth of people with NAFLD have advanced from simple steatosis to nonalcoholic steatohepatitis (NASH), and from NASH to hepatocellular carcinoma (HCC) and cirrhosis. The condition has caused sustained economic losses, but additionally caused a decline in cases’ quality of life [1]. NAFLD’s prevalence is 25.24% and 29.2% worldwide and in China, respectively. NAFLD patients are also more prone to metabolic disorders [2]. Currently, the main treatment methods of NAFLD are lifestyle intervention and drug treatment, but drug therapy has some adverse effects, such as aggravating liver injury, and there is no specific drug [3].

Mitochondria are relatively independent organelles in cells, with double-layer membrane structure and independent DNA. Mitochondria produces ATP through the process of oxidative phosphorylation on respiratory chain [4]. The transcription factor peroxisome proliferator-activated receptor α (PPARα) expression is high within hepatocytes, regulating the genes that are associated with the de novo generation and oxidation of fatty acids [5]. By controlling the expression of transcription factors taking part in mitochondrial biosynthesis, peroxisome proliferator-activated receptor gamma co-activator 1α (PGC-1α) is crucial to fatty acid oxidation and other metabolic activities inside the mitochondria [6, 7]. Fenofibrate is a fibrate drug, as a PPARα agonist, it is widely used to lower lipids against dyslipidemia [8]. However, the mechanism of fenofibrate affecting lipid accumulation and mitochondrial function in NAFLD remains to be additionally clarified.

The goals of this investigation are to (1) elaborate on whether fenofibrate regulates liver lipid accumulation by mediating mitochondrial function, and (2) explore the mechanism of PGC-1α mediated mitochondrial function to regulate lipid accumulation in hepatocytes, and provide theoretical basis and reference for finding new targets for NAFLD treatment.

## Materials and methods

### Animal experiments

#### NAFLD mice model

**Fig. 1:**
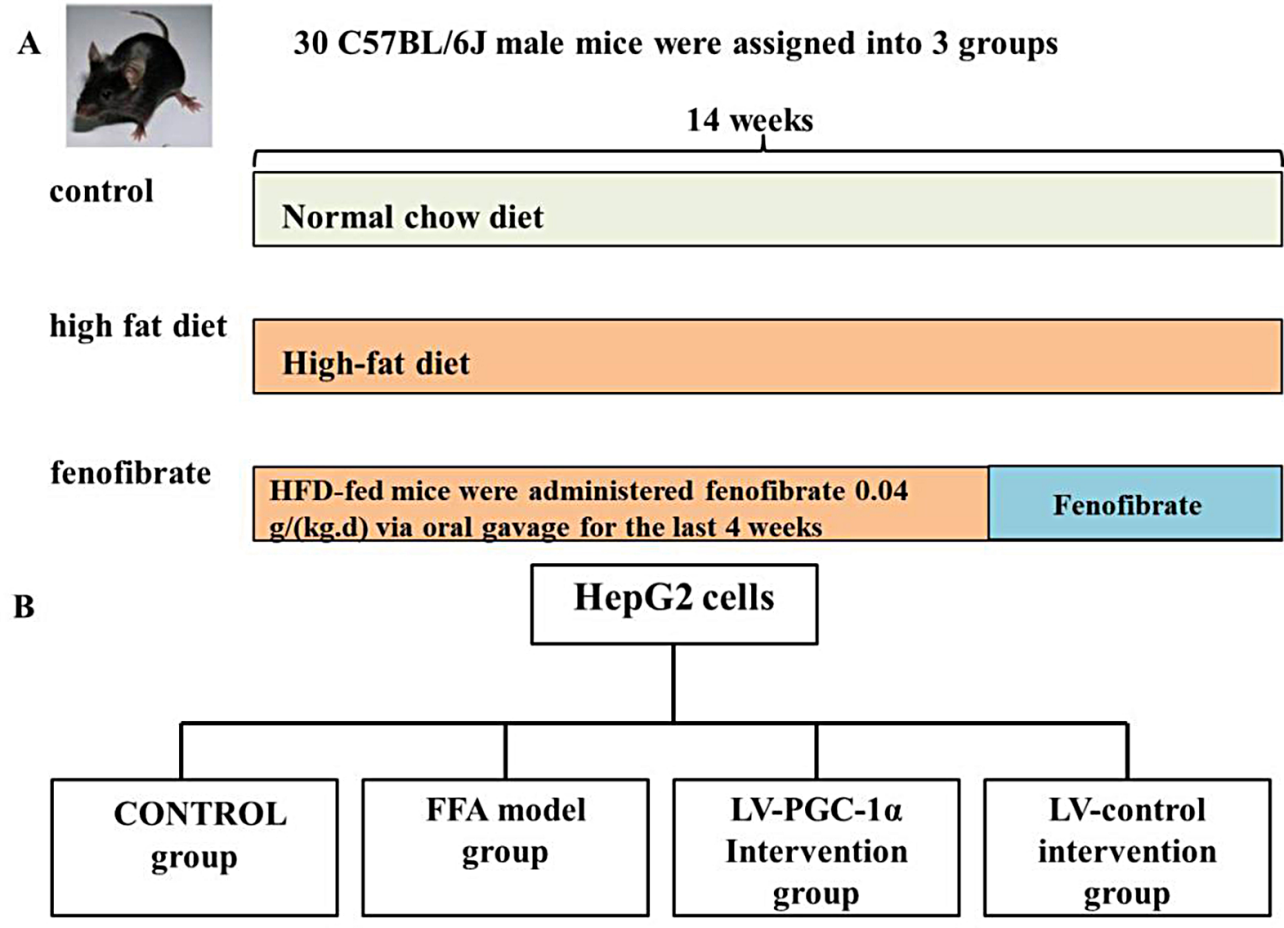
Experimental groups. (**A**) Experiment in vivo. (**B**) Experiment in vitro

Thirty male C57BL/6J mice, aged six weeks, weighing 19-25 g, could be obtained from Beijing Vital River Laboratory Animal Technology Co., Ltd., under the company’s SCXK (Beijing) 2021-0006 manufacturing license. Mice were bred at Xi’an Jiaotong University’s Experimental Animal Center, SYXK (Shaanxi) 2020-005, specified constant environment and fed normal diet, temperature (22 ± 2) ℃, humidity 50-60%, 12 h circulating lighting, noise, etc., which meet international requirements, and mice can eat and drink freely. A random allocation of male 6-week C57BL/6J mice into three groups was done (Fig. 1A), including controls, model (14 weeks of a high-fat diet), and fenofibrate [similar to the model one with administered 0.04 g/(kg.d) fenofibrate by gavage at 11 weeks for 4 weeks] groups, which contained 10 mice each. After one week of adaptive feeding, normal chow (research diets 12,450) was considered for controls, and high-fat diet (60% fat, research diets D12492, Ruidi Biotechnology Shenzhen Co., Ltd.) for 14 weeks was considered for the other two groups. For the fenofibrate group, fenofibrate capsule (France RECIPHARM FONTAINE, specification 0.2 g×10 capsules, lot number 32,804); the dose of drug intervention was 10 times of clinical adults. The tolerance of animals is greater than that of humans. In this study, equivalent dose ratio between mice and humans is about 10 times. Body weight was measured once a week throughout the study. Criteria for successful model: three mice were randomly selected, and the hepatic fat content was > 5% of the wet weight of the liver. Hematoxylin-eosin (HE) staining of the liver was performed. Under the microscope, more than 1/3 of the hepatocytes showed vacuolar degeneration, indicating that the modeling was successful. All experimental studies as approved by the Ethics Committee of Xi’an Medical College (permit No. XYLS2019001). All animals’experiments were approved by science faculty and all are based on the approved guidelines and are by ARRIVE (Animal Research) Reporting of In vivo Experiments guideline. All experimental is carried out compliance with ARRIVE guidelines.

#### Determination of liver tissue, blood biochemical indicators and oxidative stress indicators

This study record body weight once a week. At the endpoint of the study, intraperitoneal injection of 100 mg/kg pentobarbital sodium into mice for anesthesia, blood samples were obtained through cardiac puncture, then serum was separated and liver tissues were collected. Liver index=(liver wet weight/body weight)×100%. Formaldehyde-preserved liver tissue was stored for further pathological analysis. Put the fixed liver tissue block into alcohol dehydration at gradient concentration, embed it in paraffin, cut it into thin slices with thickness < 8 μm, conduct routine HE staining. Oil red O staining: frozen section thickness = 8 μm. Dye with oil red O, operate as per the instructions of the kit (Nanjing Jiancheng Technology Co., Ltd), take photos under the microscope (Leica, Germany) after staining and sealing.

Serum high-, and low-density lipoprotein (HDL and LDL), triglyceride (TG), superoxide dismutase (SOD), total cholesterol (TC), alanine and aspartate aminotransferase (ALT and AST) serum activities, and malondialdehyde (MDA) content within mice were measured.

#### Determination of ATP content, ROS content and mitochondrial membrane potential

Mitochondrial membrane potential (Δ*Ψ*_m_), and ROS and ATP contents commercial kits (Biyotime, China) were operated according to the instruction. For ATP content, part of liver tissue was washed with PBS and crushed by a homogenizer. Tissues were homogenized with cell lysate, and a protein mixture was obtained by centrifugation. ATP concentrations were reported in units of nmol/g protein.

Flow cytometer had been used to measure the level of ROS content and Δ*Ψ*_m_ of hepatocytes. Part of the fresh liver tissue was taken, cut by ophthalmic scissors, and the liver homogenate was filtered with 300 mesh nylon mesh to obtain the liver single cell suspension, which could be centrifuged for 3 min at 500–800 r/min to prepare 0.5 ml suspension containing 10^7^ cells. Flow cytometry could be utilized for evaluating the single cell suspension. There is a red fluorescence from the JC-1 polymer, and a green fluorescence from the JC-1 monomer. Δ*Ψ*_m_ level was shown by the red/green JC-1 spectral ratio. Reactive oxygen detection uses fluorescent probe DCFH-DA to detect the level of reactive oxygen species in DCF. Detect DCF with FITC parameter settings.

### Cell experiments

#### Culture conditions

The HepG2 cell line was donated by the Pathology Teaching and Research Section of Xi’an Medical College of China. 10% fetal bovine serum (Gibco, USA) was dissolved in DMEM high glucose medium and cultured in CO_2_ cell incubator. All methods were performed in accordance with the relevant guidelines for standard operating procedure and regulations.

#### Cell transfection with lentivirus

When HepG2 grows to 50–70% fusion degree, lentivirus LV-PPARGC1A (2 × 10^8^ TU/ml); Negative control virus LV-control (CON238, uBI-MCS-SV40-EGFP-IRES-puromycin, 1 × 10^9^ TU/ml), add virus infection enhancer HitransG A&P, and observe the transfection effect after 72 h. Human overexpression lentivirus vector PPARGC1A, NM_001330751 and the negative control virus (LV-control) were purchased from Shanghai Genechem Co., LTD. Detection of PGC-1α by qRT-PCR overexpression, primer sequence.

Forward: 5′-CAGAGAGTATGAGAAGCGAGAG-3′, Reverse: 5′-AGCATCACAGGTATAACGGTAG-3′,

GAPDH is an internal reference gene. The primer sequence is Forward: 5′- GCAATTCCATGGCACCGT-3′, Reverse: 5′- TCGACCCCATTGATTTGG-3′.

#### Establishment of a cell lipid loading model

Palmitic acid (PA, Sigma, USA) and Oleic acid (OA, Sigma, USA) were dissolved with DMSO in the ratio of 2:1 (concentration 0.66: 0.33 mol/L) to make free fatty acid (FFA). In vitro, four groups based on HepG2 cells were generated (Fig. 1B), including controls, the FFA model (1.5 mmol/L FFA incubation for 24 h), LV-PGC-1α intervention (similar to the FFA model one after PPARGC1A lentivirus transfection), and LV control intervention (similar to the FFA model one after negative control lentivirus transfection) groups.

#### Detection of lipid levels

The total proteins were extracted in RIPA buffer on ice from the cells or from liver tissue homogenate. The BCA method was used to quantify the protein content. The content of TC and TG was expressed as mmol/g protein. Lipid buildup inside cells had been evaluated by oil red O staining. The expression folds of lipids were determined by the absorbance value at 510 nm.

#### Mitochondrial morphology determination

Mito-tracker Green commercial kits (Biyotime, China) is a mitochondrial specific fluorescent probe. Follow the instructions, then observation with laser confocal microscope. MiNA of FIJI-ImageJ software was used, and calculated the various parameter indicators of the mitochondrial network.

#### Determination of mitochondrial ATP content and mitochondrial DNA (mtDNA) replication fold

Cellular ATP was measured via an ATP detection kit; the procedure followed the same stages previously reported. The DNA extraction kit was used to extract cell DNA, measure the DNA concentration, detect the replication fold of NADH dehydrogenase subunit 1 (ND1). ND1 gene represents the replication fold of mitochondrial DNA, and GAPDH is an internal reference gene. Configuration 20 µL reaction system for DNA amplification, using 2^−ΔΔct^ methods. ND1 primer sequence Forward: 5′-GGAGTAATCCAGGTCGGT-3′, Reverse: 5′-TGGGGTACAATGAGAGAGTAGG-3′;

GAPDH primer sequence Forward: 5′-AAGGGGAGGGGGGGGGTGT-3′, Reverse: 5′-TCAAGGGGGGGGGAAGCAG-3′. The primer were purchased from Shanghai Sangon Co., Ltd.

#### Western blot

RIPA was utilized for lysing the protein in the ice-cold supernatant, and the BCA assay was used to measure the concentration. SDS-PAGE was used to separate and transfer 40 µg of boiling, denaturated protein to nitrocellulose membranes. Sealed for 1 h, incubated of primary and secondary antibodies, and fix it with ECL imaging. The absorbance values were analyzed via Image J. GAPDH was the internal reference protein. Expression of PPARα, PGC-1α, NRF-1, TFAM and GAPDH protein were detected with the following primary antibodies: PPARα rabbit polyclonal antibody, PGC-1α rabbit polyclonal antibody (Abcam Biotechnology, USA), TFAM Rabbit Polyclonal Antibody (Biyotime, China), and GAPDH rabbit monoclonal antibody (Cell Signaling Technology, USA). The secondary antibody was goat anti rabbit kit (Beijing Zhongshan Jinqiao Biotechnology Co., Ltd, China).

### Statistical analysis

SPSS 22.0 was employed for analyzing data. The findings were presented as mean ± standard deviation (SD). One-way ANOVA had been utilized for comparison of data between different groups, and there was a significant difference among groups when the difference was *P* < 0.05. GraphPad Prism 6 was used for drawing figures.

## Results

### High-fat diet with fenofibrate treatment reduced liver steatosis, body weights, and liver indexes

**Fig. 2:**
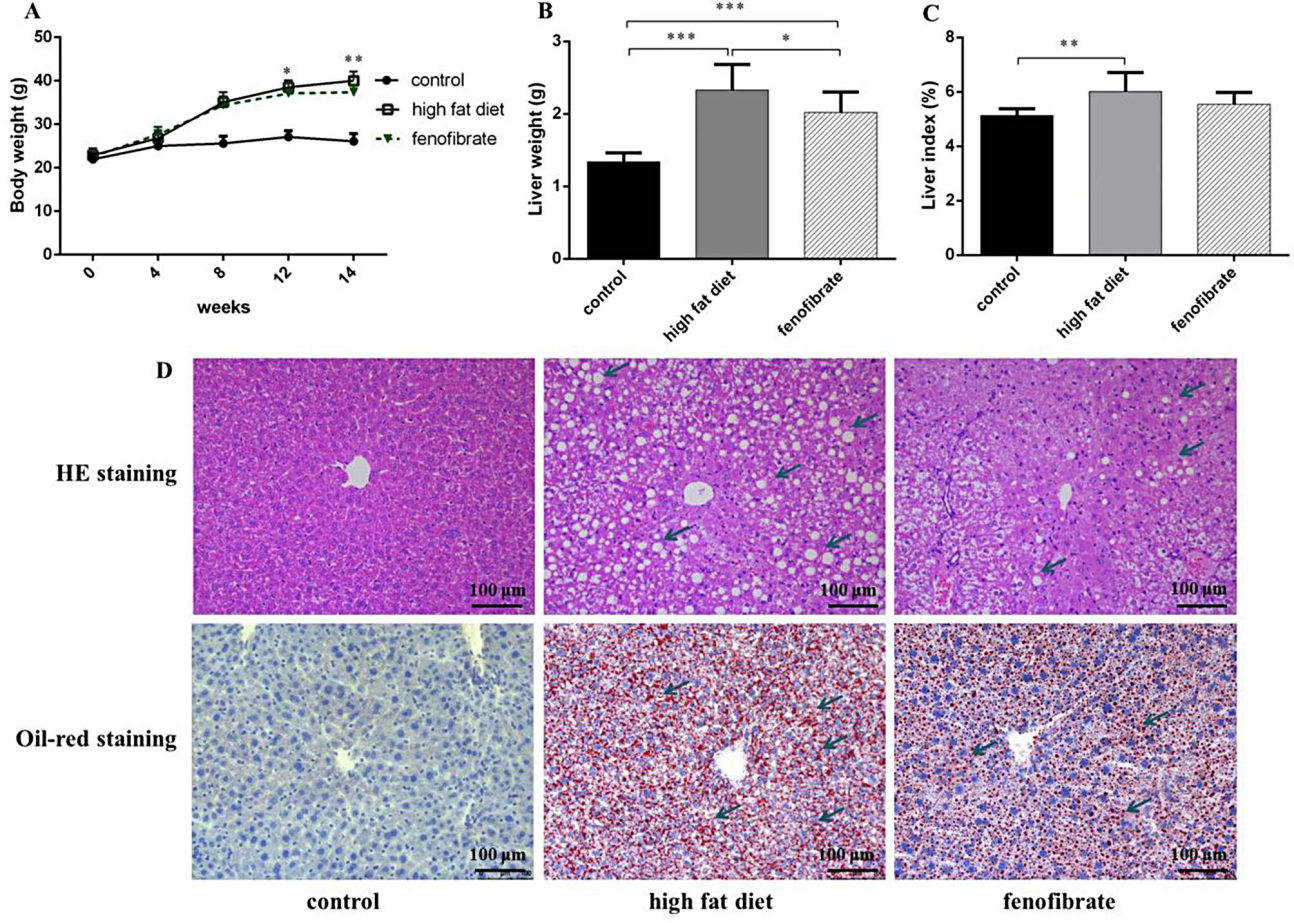
Changes of body weight, liver weight and liver index in mice. (**A**) Body weight. (**B**) Liver weight. (**C**) Liver index. (**D**) The morphology the liver stained by HE staining and oil red O staining×200. (*n* = 10). *Notes*: ^*^P < 0.05; ^**^P < 0.01; ^***^P < 0.001

The liver weight and index within the high-fat dietary increased more remarkably than controls, 2.33 ± 0.36 g vs. 1.34 ± 0.12 g and 6.01 ± 0.71 (%) vs. 5.13 ± 0.25 (%), respectively. Furthermore, liver weight reduced significantly, the liver index decreased in the fenofibrate group, but no remarkable difference with the high-fat dietary animals was observed. The results show that fenofibrate slowed down lipogenesis and weight gain. The morphology and structure of the liver tissue was sound without steatosis in the controls. A lot of fat droplets were observed in the high-fat dietary animals’ hepatocytes. HE staining showed obvious damage to liver tissue in the high-fat dietary mice, hepatic fatty degeneration was scattered throughout the liver. Fenofibrate reduced the pathological state of fatty degeneration in the liver (Fig. 2).

### Fenofibrate treatment reduced the lipids levels and oxidative stress, exacerbated mitochondria dyfunction in mice

**Fig. 3:**
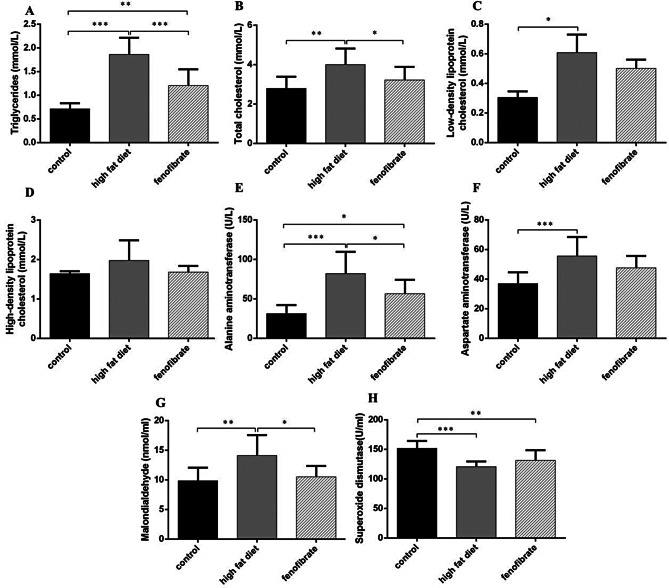
Biochemical indexes of mice in serum. (**A**) TG level. (**B**) TC level. (**C**) LDL-C. (**D**) HDL-C. (**E**) ALT activity. (**F**) AST activity. (**G**) MDA content. (**H**) SOD activity. (*n* = 10). *Notes*: ^*^P < 0.05; ^**^P < 0.01; ^***^P < 0.001

**Fig. 4:**
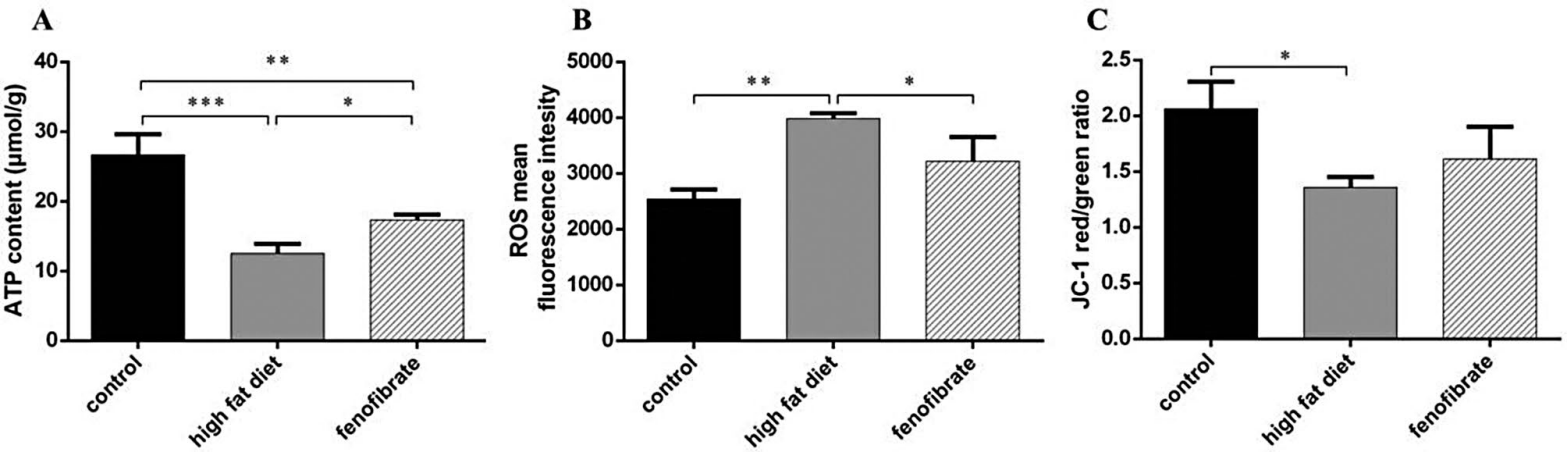
Mitochondrial function index in hepatocytes. (**A**) ATP content. (**B**) ROS mean fluorescence intensity. (**C**) Mitochondrial membrane potential, JC-1 red/green fluorescence ratio. (*n* = 3). *Notes*: ^*^P < 0.05; ^**^P < 0.01; ^***^P < 0.001

LDL-C, TG, TC, AST, ALT values and MDA content, and SOD activity in serum diminished with high-fat diet. TG and TC decreased more remarkably in the fenofibrate than model group. The results suggested that fenofibrate improved liver function, reduced liver injury, and reduced serum TG and TC; the content of MDA decreased, the SOD activity increased, suggesting that fenofibrate reduced the oxidative stress (Fig. 3). High-fat diet reduced ATP content and Δ*Ψ*_m_, increased ROS content in hepatocytes, while fenofibrate alleviated the injury (Fig. 4).

### Lentivirus transfected HepG2 cells

**Fig. 5:**
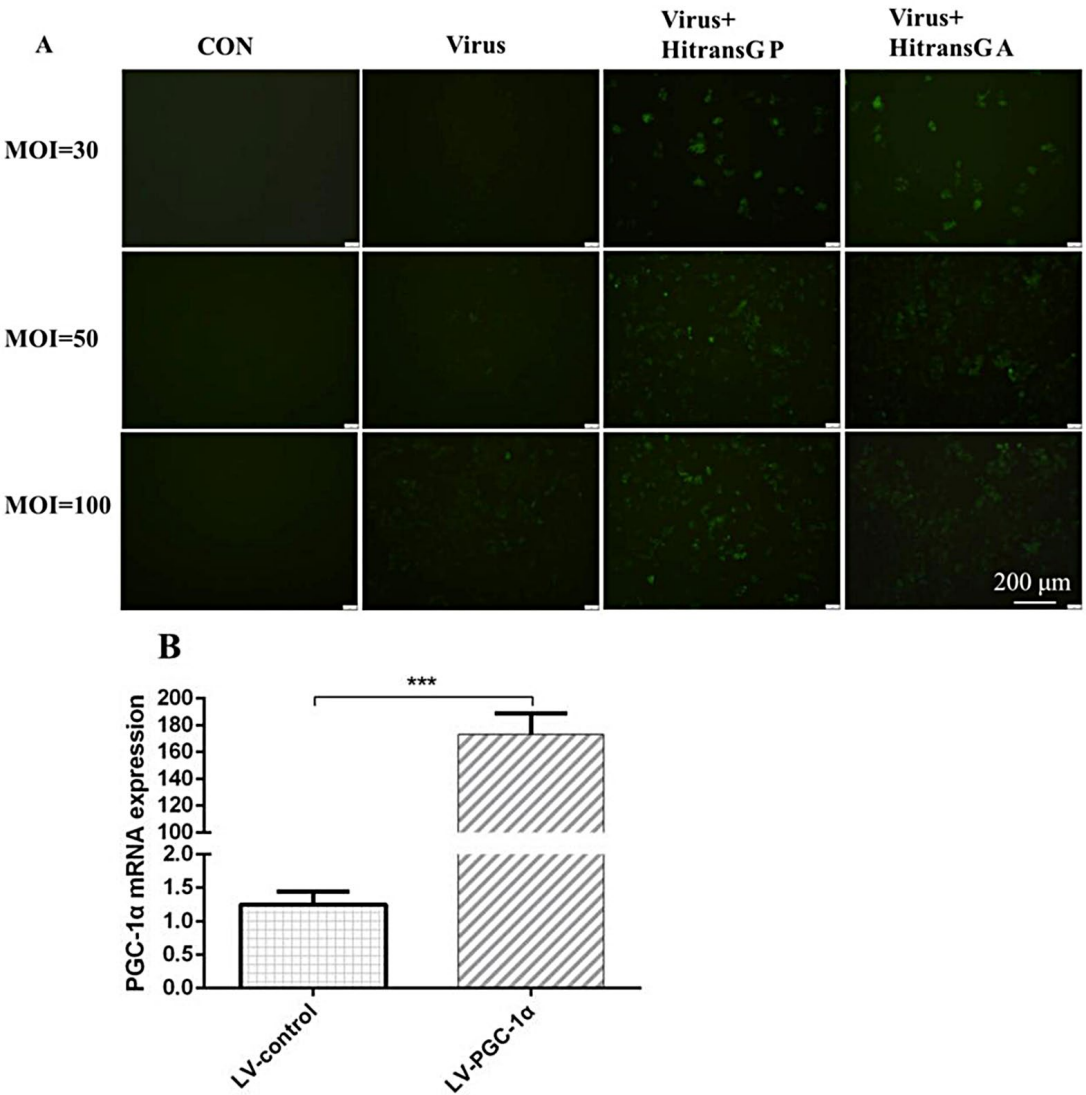
Transfection efficiency of lentivirus. (**A**) Lentivirus transfected HepG2 cells for 72 h (×100). (**B**) PGC-1α gene overexpression. *Notes*: ^***^P < 0.001

In LV-PGC-1α intervention group, the fluorescence intensity increased with the transfection efficiency. When MOI = 100 and 72 h, the transfection efficiency of virus with P-type transfection enhancer was the highest group, and MOI = 50 was close to the transfection efficiency of MOI = 100, so MOI = 50 with P-type transfection enhancer was selected, 72 h was selected as the experimental endpoint. Additionally, PGC-1α overexpressed significantly in LV-PGC-1α intervention group more than HepG2 cells (Fig. 5).

### PGC-1α overexpression reduced the lipid accumulation in HepG2 cells

**Fig. 6:**
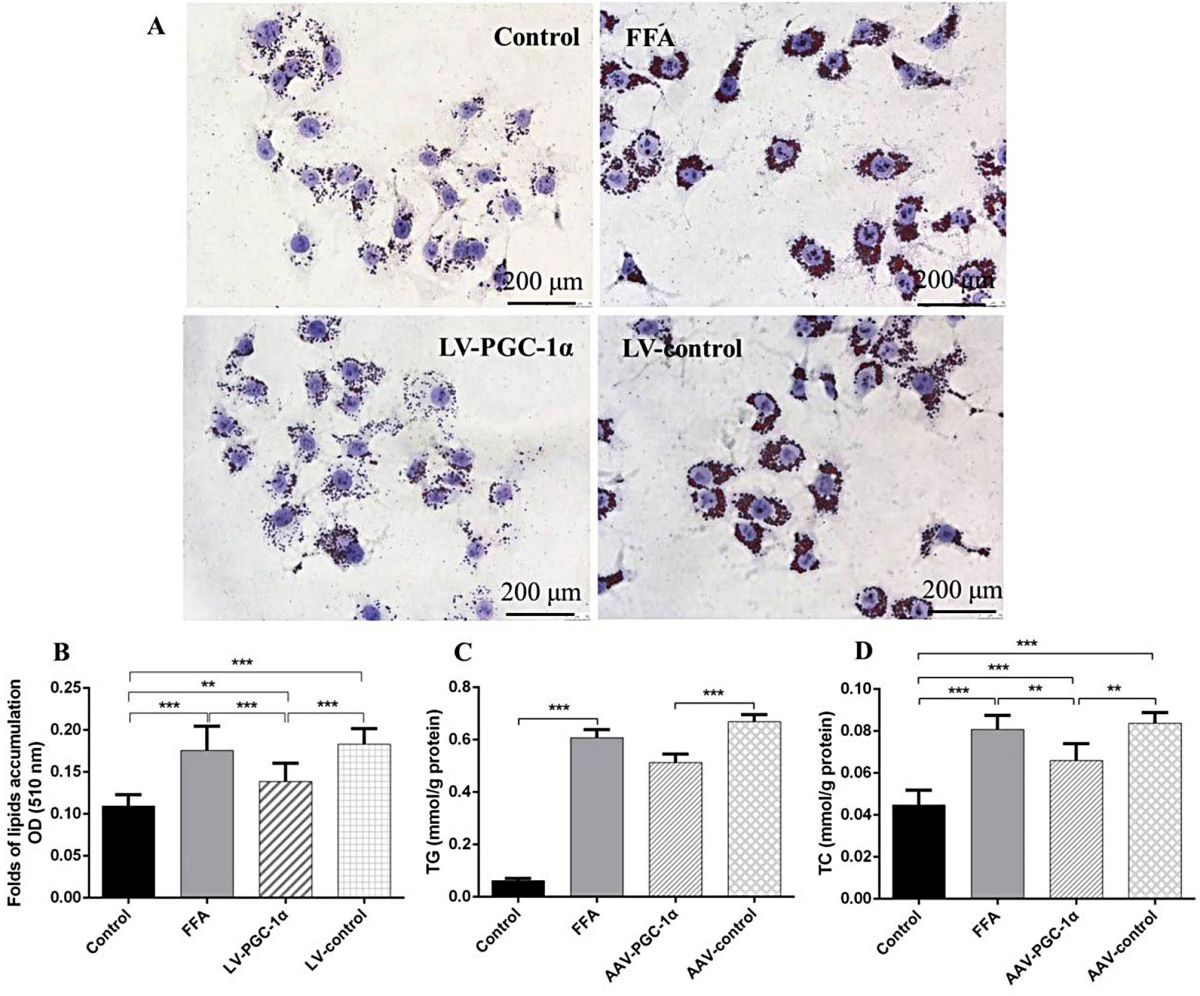
PGC-1α overexpression attenuated lipid accumulation induced by FFA in HepG2 cells. (**A**) Oil red O staining, the magnification of each panel is ×400. (**B**) Folds of lipids accumulation OD at 510 nm. (**C**) Intracellular TG (**D**) Intracellular TC. (*n* = 3). *Notes*: ^*^P < 0.05; ^**^P < 0.01; ^***^P < 0.001

The difference in absorbance values at 510 nm between LV-control intervention group and LV-PGC-1α intervention group was significant. Furthermore, LV-PGC-1α intervention significantly reduced intracellular TG and TC content (Fig. 6), indicating that PGC-1α overexpression inhibited the lipid accumulation in HepG2 cells.

### FFA induces excessive mitosis into fragmented in mitochondrial morphology

**Fig. 7:**
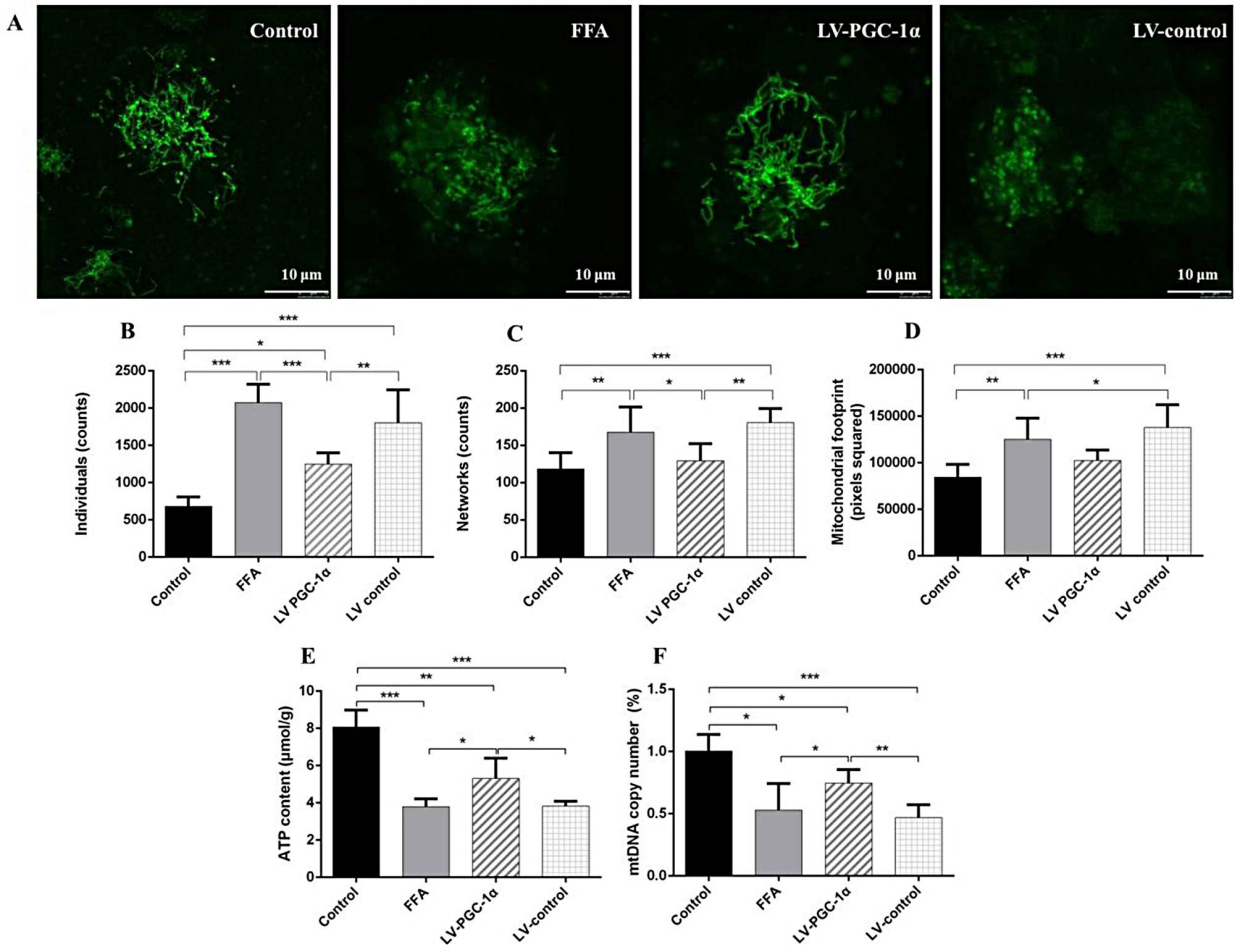
Mitochondrial morphological and functional indexes of HepG2 cell. (**A**) Mito-tracker fluorescent probe was used to detect the mitochondrial morphological changes of cells. (**B**–**D**) Morphological characteristics of mitochondrial individuals, number of networks, mitochondrial footprint. (**E**) ATP content. (**F**) mtDNA replication number. (*n* = 3). *Notes*: ^*^P < 0.05; ^**^P < 0.01; ^***^P < 0.001

The morphology and network connectivity of cell mitochondria were observed, FFA induces excessive mitosis into fragmented mitochondria (Fig. 7A). Compared with the control group, the total mitochondrial area was significantly increased in the FFA model group cells, with more than double the number of punctate and rod-shaped mitochondria, and a significant increase in the number of reticular mitochondria. Compared with the LV control intervention group, the total mitochondrial area significantly reduced, the number of punctate and rod-shaped granules mitochondria, reticular mitochondria decreased significantly in LV-PGC-1α intervention group (Fig. 7B–D).

### PGC-1α overexpression improved mitochondrial functional related indexes including ATP content and mtDNA replication number in HepG2 cells

There were significant differences in ATP content and mtDNA replication number between groups. Compared with control group, the ATP content reduced significantly in FFA model group, 3.80 ± 0.42µmol/g vs. 8.08 ± 0.89µmol/g (*P* < 0.001). ATP content elevated in LV-PGC-1α intervention group than LV-controls, 5.30 ± 1.10µmol/g vs. 3.82 ± 0.26µmol/g (*P* < 0.05, Fig. 7E). The mtDNA replication number in FFA model reduced remarkably more than controls and increased more remarkably with LV-PGC-1α than LV-controls (Fig. 7F). PGC-1α overexpression may ameliorate mitochondrial biosynthesis.

### Mice with high-fat diet decreased PPARα and PGC-1α proteins expression, fenofibrate reduced the injury

**Fig. 8:**
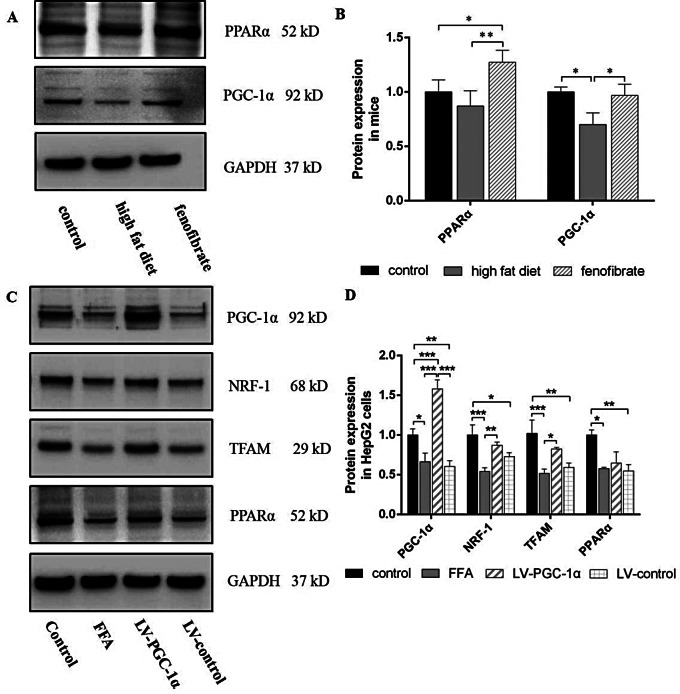
PPARα, PGC-1α, NRF-1 and TFAM protein expression. (n = 3). (**A**, **B**) Protein expression in mice. (**C**, **D**) Protein expression in HepG2 cells. *Notes*: ^*^P < 0.05; ^**^P < 0.01; ^***^P < 0.001

PPARα and PGC-1α expression remarkably diminished more in the high-fat dietary group than controls. Moreover, fenofibrate activated PPARα expression, increased PGC-1α expression. Fenofibrate reduced lipid accumulation by up-regulating mitochondrial biosynthesis (Fig. 8A, B). PGC-1α overexpression increased the PPARα, NRF-1 and TFAM expression in HepG2 cell more than LV-controls. These results confirmed that PGC-1α overexpression inhibited lipid accumulation by increasing NRF-1 and TFAM expression (Fig. 8C, D).

## Discussion

**Fig. 9:**
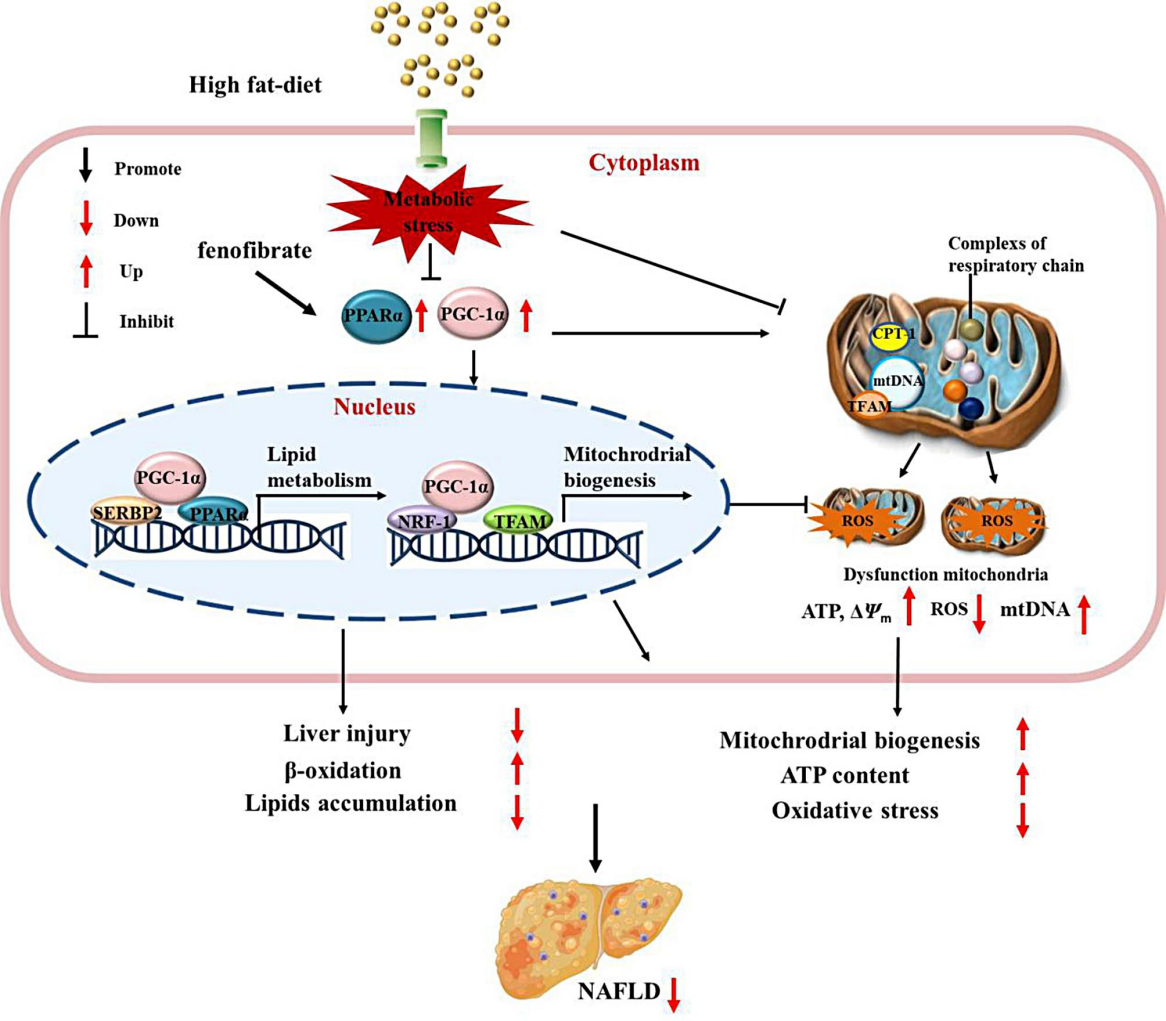
Mechanism of fenofibrate alleviates NAFLD by enhancing the expression of PPARα/PGC-1α

Due to the systemic effects of metabolic disorders, NAFLD pathology impairs lipid metabolism and insulin sensitivity, increases oxidative stress and systemic inflammation, exacerbates liver lipid deposition, and cardiovascular complications [9, 10]. The liver of NAFLD patients accumulates fat in the form of TG, which mainly comes from the esterification of FFA and others [11, 12]. In this study, NAFLD mice model was established through high-fat diets. PGC-1α related mitochondrial function was investigated in NAFLD cell model stimulated by FFA. Functionally, fenofibrate treatment lowered blood lipids, limited the lipid droplet deposition and reduced hepatocyte oxidative stress. Liver pathology, and mitochondrial function indicators remarkably differed between the model and control groups, fenofibrate improved objective indicators. Mechanistically, fenofibrate up-regulated PPARα/PGC-1α signaling pathway, promoted mitochondrial biosynthesis, reduced oxidative stress damage and lipid accumulation of liver. PGC-1α overexpression enhanced mitochondrial biosynthesis, ATP production, reduced oxidative stress, strengthened the oxidative decomposition of fatty acids, and reduced the lipid accumulation in HepG2 cells (Fig. 9). The replication methods of NAFLD animal models include high-fat diet (20% carbohydrates, 20% protein, 60% fat), high-fructose diet, methionine and choline deficiency diet, high cholesterol diet, genetic defects, and other methods [9, 13]. This study’s animal model replication strategy involves maintaining a 14-week, high-fat diet, which is similar to the diet of human NAFLD patients and better simulates the etiology, pathogenesis, and pathological changes of human NAFLD. In other NAFLD animal model studies [14, 15], there are also similar experimental results. For example, the liver tissue steatosis caused by high-fat diet had obvious inflammatory changes, the activity of SOD enzyme decreased significantly in liver tissue homogenate, and the serum TG, TC and LDL increased significantly. These results suggested that the metabolic disorder of energy load is an important cause of NAFLD, and the modeling method of high-fat induction is relatively stable.

Fenofibrate is a lipid-lowering drug with clear effects. Fenofibrate communicates with PPARα, activates PPARα related signaling pathways mediate adipocyte proliferation, regulates body fat metabolism, facilitates fat redistribution, avoids excessive accumulation of liver fat, and improves non-alcoholic fatty liver disease. Fenofibrate inhibits the metabolism of lipid and protein hydrolytic enzymes, alleviates bile stasis, and alleviates the progression of liver fibrosis. It was used to affect other microvascular vascular complications of diabetes, such as improving diabetes kidney damage, diabetes retinopathy, diabetes neurological complications, etc. It also was used to alleviate vascular diseases caused by hypertension, such as hypertensive nephropathy, coronary heart disease, to slow down arteriosclerotic diseases, such as coronary atherosclerosis, stroke, peripheral vascular disease of limbs, etc. [16]. The pathological histology analysis result showed that there were some morphological abnormalities and characteristic pathological change in liver, together with the presence of different lipids, like cholesterol esters, and triglycerides [17, 18]. Lipid buildup is lowered by the simultaneous actions of free fatty acid import from peripheral adipose tissue and lipid export through very low density lipoprotein particles from the liver [19]. This study focuses on the accumulation of liver triglycerides and abnormal changes in blood lipids caused by excessive lipid intake, without directly detecting the lipid transport of very low density lipoprotein particles. Meanwhile, when a large amount of free fatty acids flood into liver and reach a peak, excess lipids lead to lipotoxicity. Lipotoxic stress eventually damages mitochondria and endoplasmic reticulum, leading to hepatocytes death and inflammation [20].

The energy metabolism of hepatocytes is vigorous, with a number of mitochondria ranging from 1000 to 2000, accounting for 20–40% of the cell volume. There are double membrane structures in mitochondria. From the outside to the inside, there are cell outer membrane, inner membrane and matrix. The inner membrane folds into a ridge structure, and cellular respiration chains are distributed on the ridge. The hydrogen and electron transmitters on the cellular respiration chain, as well as the respiratory enzyme complex, use energy materials to complete oxidative phosphorylation to produce ATP by transferring electrons [21]. The mitochondrial membrane potential, denoted by the symbol Δ*Ψ*_m_, results from the bidirectional distribution of protons between the mitochondrial inner and outer membranes. A reduction in Δ*Ψ*_m_ indicates that the cell is in the early stages of apoptosis. Oxidative phosphorylation is the source of ROS, and the mitochondria are the primary target organ for ROS. The structure and function of proteins, nucleic acids (DNA/RNA), and lipids can be damaged by continuous oxidative stress [22]. Common mitochondrial dysfunction includes increased ROS production, decreased ATP content due to oxidative phosphorylation, decreased mtDNA biosynthesis, abnormal mitochondrial morphological fusion and division imbalance, autophagy damage, etc. [23]. MtDNA is an independent double stranded DNA, which is more susceptible to ROS attacks due to the lack of DNA repair enzymes [24]. Firstly, ROS attacks mtDNA and causes oxidative damage, which destroys the structure and function of cellular proteins and DNA/RNA through the formation of oxygen free radicals. 8-hydroxydeoxyguanine nucleoside is a molecular marker of mtDNA damage. Oxidative stress accelerates cell membrane lipid peroxidation, during which MDA content increases together with an after-consumption reduction in SOD and GSH-PX antioxidant enzymatic activities [25]. Secondly, PGC-1α regulates the mtDNA biosynthesis. When PGC-1α is down-regulated, the copy number of mtDNA decreases, the rate of oxidative phosphorylation slows down, and the ATP content decreases [26]. Mitochondrial DNA transcription, replication, and translation all began when PGC-1α and NRF-1 co-transcriptionally activated the mitochondrial transcription factor A (TFAM) [27]. In this study, under high-fat stress, the ROS content of hepatocytes increased, mitochondrial Δ*Ψ*_m_ and ATP content decreased. Fenofibrate increased ATP content in hepatocytes, reduced ROS production, and inhibited decreasing of Δ*Ψ*_m_. It alleviated mitochondrial dysfunction. The interaction of PGC-1α and PPARα induced increasing of the number of mitochondria and enhancing β-oxidation function. Besides, PGC-1α activation enhances mitochondrial antioxidant enzymes expression and alleviates oxidative stress in HepG2 cells.

Transcription factors such as PPARα, sterol regulatory element binding proteins, etc. are activated by ligands in genetic networks that control lipid metabolism through nuclear hormone receptors [28]. Both transcription factors and intracellular receptors, nuclear hormone receptors play an important role in regulating gene expression by binding certain lipid molecules. Sterol regulatory element binding protein-1c regulates genes involved in fatty acid production. The decomposition pathway of liver lipids involves fatty acid oxidation, which transports TG out of the liver with extremely low density lipoprotein [29]. Any abnormality in the absorption, synthesis, and decomposition process of lipid metabolism may lead to excessive accumulation of liver lipids. Fenofibrate, a PPAR agonist, is often used for dyslipidemia. The possible mechanism is that fenofibrate activates PPARα, prevents excessive accumulation of liver TG [30, 31]. Upregulating PPARα expression can promote mitochondria transcriptional regulation of key β-oxidation genes. The genes accelerate the oxidation rate of fatty acids and inhibit steatosis of liver tissue caused by lipid accumulation in hepatocytes. In addition, PPARα has anti-inflammatory effects and can enhance the activity of fibroblast growth factor 21 and reduce nuclear factor kappa B activity [32]. PGC-1α is a downstream molecule of PPARα, high expression of PGC-1α promotes mitochondrial biosynthesis, thereby promotes free fatty acids β-oxidation [33]. NAFLD model mice given a high-fat diet showed substantial reductions in the expression of PPARα and PGC-1α in the liver. Fenofibrate upregulated PPARα and the mitochondrial biosynthesis related molecule PGC-1α, reduced oxidative stress damage and accumulation of lipids within the hepatocytes, and slowed down NAFLD pathology. PGC-1α overexpression enhanced mitochondrial biosynthesis, ATP production, reduce oxidative stress, strengthen the oxidative decomposition of fatty acids, and reduced the lipid accumulation of HepG2 cells. Similarly, other relevant research reports have also shown that a high-fat diet leads to an increasing in TG synthesis in mouse liver, downregulation of key molecules related mitochondrial biosynthesis, and relatively insufficient oxidation and utilization of fatty acids in liver, exacerbate the progression of NAFLD [34, 35]. Furthermore, fenofibrate helps reduce inflammation, prevents blood clots, and enhances endothelial function [36]. This study used FFA to induce the formation of a NAFLD cell model, the lipid accumulation of PGC-1α intervention group is reduced by enhancing mitochondrial biosynthesis, ATP generation, fatty acid oxidation and decomposition.

## Conclusions

There were several strengths in current research. Firstly, the study further explored the fenofibrate improving mitochondrial function by enhancing expression of PPARα/PGC-1α. Secondly, further research on the issue of mechanism were conducted by regulating PGC-1α overexpression. However, this study also had limitations. Although the factor PPARα, PGC-1α, NRF-1 and TFAM related to mitochondrial biosynthesis and lipid metabolism were explored, it is necessary to explore the expression of other transcription mediators involved in liver lipid metabolism and synthesis comprehensively and systematically. The liver, as the core organ of human lipid metabolism, affects the lipid levels acting on the circulatory system by regulating the metabolism of TG and TC, and deeply discusses the regulatory mechanism of liver mitochondria for lipid, which has important reference value for hyperlipidemia, atherosclerosis and other conditions’ management.

## Data Availability

All data generated during this study are included in this published article.

## Abbreviations

ALT: Alanine aminotransferase
AST: Aspartate aminotransferase
ATP: Adenosine triphosphate
FFA: free fatty acid
GSH-PX: glutathione peroxidase
HCC: hepatocellular carcinoma
HDL: High-density lipoprotein
HE: Hematoxylin and eosin
HFD: High-fat diet
HRP: horseradish peroxidase
LDL: Low-density lipoprotein
MDA: Malondialdehyde
mtDNA: mtiochondrial DNA
NAFLD: Nonalcoholic fatty liver disease
NASH: nonalcoholic steatohepatitis
ND1: NADH dehydrogenase subunit 1
NRF1: Nuclear respiratory factor 1
OA: Oleic acid
ROS: Reactive oxygen species
SOD: Superoxide dismutase
PA: palmitic acid
PGC-1ɑ: Peroxisome proliferator-activated receptor γ co-activator 1α
PPAR-ɑ: Peroxisome proliferator-activated receptor-ɑ
qRT-PCR: Quantitative real-time polymerase chain reaction
TFAM: mitochondrial transcription factor A
TC: Total cholesterol
TG: Triglycerides
Δ*Ψ*_m_: mitochondrial membrane potential
